# Tweaking of radiation and chemotherapy schedules is the new normal during the COVID-19 crisis: perspective from oncologists at a tertiary care health institute

**DOI:** 10.3332/ecancer.2021.1177

**Published:** 2021-01-22

**Authors:** Sandip Kumar Barik, Sovan Sarang Dhar, Saroj Kumar Das Majumdar, Dillip Kumar Parida

**Affiliations:** Department of Radiation Oncology, All India Institute of Medical Sciences, Bhubaneswar 751019, Odisha, India

**Keywords:** COVID-19, cancer, treatment, radiotherapy, chemotherapy, patient care

## Abstract

Patients with cancer are at a higher risk of infection with Severe Acute Respiratory Syndrome Coronavirus-2 (SARS-COV-2) than the general population. In India, it has become a significant health problem of utmost importance, and India’s Government has issued health advisories. Lockdown brought many unforeseen problems for patients and hospitals, leading to confusion and chaos. The aim of this article is to identify various issues related to our hospital, follow-up, nutrition, treatment and psychosocial issues. Multiple changes were made in the hospital, departmental and treatment policy for cancer patients’ convenience and safety. As India is in the peak of COVID-19, these types of modifications and modifications of treatment schedules will be the ‘New Normal’.

## Introduction

The 2019 novel coronavirus’s appearance has been shortly followed with global spread and rise in cases to such an extent that the World Health Organization (WHO) declared the outbreak as public health emergency of international concern on 30 January 2020 [1]. The outbreak reached more than epidemic proportions in a short period, and nearly 70 days after the first report of this virus, the WHO announced COVID-19 as a pandemic on 11 March 2020. Patients with cancer are at a higher risk of infection with Severe Acute Respiratory Syndrome Coronavirus-2 (SARS-COV-2) than the general population [2] and deteriorate more rapidly than those without the disease (median time to severe events 13 days versus 43 days) [2]. Immunosuppression in cancer patients caused by either chemotherapy (CT), radiotherapy or the malignancy itself makes cancer patients more liable to be more vulnerable to COVID-19 than their counterparts [3, 4]. The majority of cancer patients are older and have one or more comorbidities, thereby increasing the risk of death from COVID-19 [5]. The rise of COVID-19 cases brought a flurry of many unforeseen problems for cancer patients, such as already immunosuppressed status, multiple preexisting comorbidities, numerous visits to the hospital and longer treatment duration. Thus, a robust and systematic policy has to be followed for the smooth functioning of the cancer facility.

### Indian perspective

The first case of COVID-19 infection in India was reported on 30 January 2020 [6]. Considering India’s vast population, the theoretical magnitude of COVID-19 can be so overwhelming that it can become unmanageable anytime. By mid-March, it had become a significant health problem of utmost importance in India, and the Government of India started issuing health advisories on COVID-19 on 11 March and 16 March 2020. Considering this, on 22 March 2020, a 14-hour nationwide voluntary ‘Janata Curfew’ was called by India’s prime minister [7]. A nationwide indefinite lockdown was imposed from 25 March 2020, almost bringing a nation of 1.3 billion to a grinding halt [8]. Till now, lockdown has been implemented in four different stages for different periods having different grades of restrictions and relaxations. As reported on 28 November 2020, India has the second-highest number of confirmed cases globally, with 9,351,224 cases and 136,238 deaths [9].

This paper intends to report the spectrum of problems and challenges the department and patients face during the pandemic and how they are dealt with.

## The spectrum of problems and COVID-19 related changes/modifications

### Effect of lockdown

Cancer treatment is usually scheduled over a period of time. Therefore, lockdown brought many travel and policy-related problems, leading to confusion and chaos among cancer patients. Inquiries flooded in and the department initiated a telemedicine facility, answering their queries over phone and via email. In India, the varying administrative rules and regulations with accompanying lockdown significantly affected the transport and movement of non-COVID-19 patients for their day-to-day necessities from healthcare providers.

**Figure 1.**
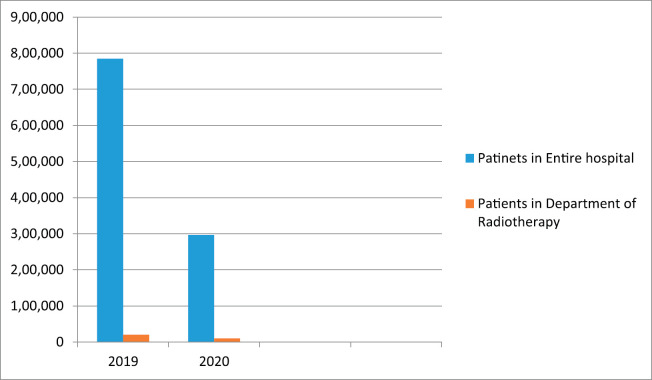
Comparing the number of patients in the outpatient’s department attended in the entire hospital and Department of Radiotherapy between March to November 2019 and 2020.

Our centre is a Tertiary Health Care Institute of National Importance in the Eastern region of India. It is a 920 bed hospital with 241 faculties working in 43 departments (34 clinical departments) towards improving healthcare, medical education and research. Figure 1 shows the comparative number of patients attending the outpatient department of the entire hospital and the Radiotherapy department from March to November 2019 and 2020. As clearly evident from the figure, there had been a drastic fall in the number of patients attending the outpatient department compared to the previous year. The number of patients attending the outpatient department for the entire institute was 7,84,765 in 2019 and 2,96,403 in 2020 (till November 2020). At the same time, the number of patients attending the Radiotherapy outpatients department was 20,365 in 2019 and 10,177 in 2020 (till November 2020). The data of patients undergoing treatment during the period of lockdown from March to November 2020 were compared with the previous year (2019) data during the same time at our institute (Table 1). The outpatient visit dropped to 10,177 patients during the lockdown period compared to 20,365 patients from the previous year (50.02% less). However, inpatient admission grew from 1,116 during the last year to 1,281 during lockdown (14.7% increase in admissions), which allowed more patients in the hospital as they had nowhere to go.

Similarly, in patients receiving External Beam Radiotherapy, the fractionation delivered was 3,998 fractions during lockdown versus 10,997 fractions during the same time a year before, showing a 63.46% drop in radiation fraction delivered. Furthermore, the number of Brachytherapy fractions delivered (123 fractions versus 183 fractions) showed a drastic decrease of 32.78% during the lockdown. During the lockdown, treatment machine occupancy dropped to 50% from 50 patients treated per day before lockdown to 25 patients during the lockdown. Daycare ward admissions for CT, blood transfusion (BT) and supportive care (SC) also dropped to 4,110 from 6,961 (40.95%) drop.

### Impact of COVID-19 on hospital daily routine in relation to:

#### Hospital policy

The Hospital has to deal with the dual problem of limited beds and limited staff due to quarantine, high-risk contact, lockdown and work rotation. Out of 920 beds, 100 beds were allotted for a separate COVID-19 unit with ICU beds and ventilation facilities (post diagnosis) and 50 beds were allocated for COVID-19 isolation unit (pre-COVID-19 diagnosis). The hospital’s basic policy for cancer patients is to prevent them from getting infected with COVID-19. Radiotherapy and oncology departments should ideally remain COVID-19 free sanctuaries [10]. In general, asymptomatic cancer patients should stay at home and avoid coming to the hospital. If they need admission for any reason, they were advised minimum stay at the hospital. There was no limitation to beds in cancer care wards but admissions were restricted because of strict COVID-19 related protocols. Asymptomatic patients were discharged from indoor wards. Patients were left stranded in the city as the lockdown was enforced. A few patients who lived far away from the city and could not go home were allowed to continue in hospital wards on the grounds of empathy. Disruption in the supply chain created an initial shortage of personal protective equipment (PPE), masks and sanitizers, which created a panic situation among health care workers (HCWs) and patients. This situation was gradually ironed out in due time.

#### Departmental policy

The onus and responsibility to stay safe and keep patients safe were significant challenges. The COVID-19 pandemic posed a considerable threat to cancer patients. To tackle these problems, major oncology societies worldwide came out with different guidelines for the effective management of cancer patients [11–16]. Translating these guidelines from paper to reality on the ground is an uphill task. Cancer patients being immune-compromised, a robust policy was required to treat and protect these patients from COVID-19 infection and complications from cancer treatment. Patients receiving antitumour therapy or surgery within 2–4 weeks of developing symptoms had a worse outcome [17, 18].

#### Impact on cancer diagnosis and workup

Early diagnosis and treatment of cancer have a definite effect on the disease’s survival. Multiple sets of diagnostic studies and laboratory tests are required for confirmation of diagnosis and staging of cancer. The need for these patients’ safety during the COVID-19 pandemic is to avoid overcrowding, following the social distancing norm during these procedures. So, patients were prioritised before ordering these tests. Asymptomatic patients who have already received treatment need not go for any routine diagnostic or laboratory tests. Only symptomatic and aggressive tumours requiring urgent intervention required these tests. During the initial lockdown period, there was a massive workload on the government-run centres, which have to deal with limited slots and limited staff. Necessary specific tests for cancer patients like positron emission tomography (PET) and bone scans stopped due to the unavailability of radionuclides as air services between states inside the country were closed.

### Modifications of cancer management

The Radiotherapy Department took measures for patients during the COVID-19 pandemic. The Radiotherapy machines, daycare CT wards, oncology ward, waiting room, and outpatient departments (OPDs) are always at very high risk for virus spread. Even one COVID-19 disease affected individual could jeopardise the lives of hundreds of cancer patients getting cancer treatment, or reversely staff getting infected, which would lead to the whole system shutting down.

#### Measures at the department level

- The COVID-19 pandemic management task force was created, responsible for formulating and implementing the standard operating procedures for the hospital from time to time, following national health policy.
- The department’s staff were sensitised, trained about the seriousness of the issue and how to effectively follow the non-touch technique, use of PPE and isolation to quarantine.
- All the staff involved in invasive procedures like Brachytherapy, treating patients with head and neck cancer, patients with tracheostomy tubes in situ during treatment are provided with PPE, following a strict sanitisation protocol.
- The department, Radiotherapy machines are sanitised twice daily, once before the commencement of treatment and once after treatment completion.
- The OPD’s schedule was changed from specific days to a daily basis for preventing overcrowding. The OPD’s shifted to a well-ventilated area instead of closed rooms.
- The patients were educated about protecting themselves from being contaminated from the disease. Patients and relatives were given handouts of do’s and don’ts to stay safe from contracting viruses. Patient education material played through Audio-Visual Sets in the waiting room.
- Social distancing, use of face cover were maintained among patients during their stay in the department. Only one attendant per patient was allowed. All patients were given a specific time slot according to their cancer sites for their radiotherapy appointment, simplifying the machine’s treatment setup process.
- The Radiation therapists were rotated on a shift basis limiting a smaller number of people getting exposed to patients.
- Regular mock drills are conducted in the department to assess the preparedness for any untoward situation.
- Wherever possible the central air-conditioning system was closed and split air conditioners were installed.

#### Modifications in the radiotherapy treatment

The modification was done based on the primary Radiation Oncology group recommendation (Tables 2 and 3). In the COVID-19 pandemic, ‘Less is the New Normal’. The primary purpose of these modifications is to decrease treatment time, respecting an effective or equivalent alternative dose to the standard schedule dose. All efforts to complete the radiotherapy treatment of patients who were currently getting radiation were prioritised when the lockdown was enforced.

##### Before starting radiotherapy:

- COVID-19 reverse transcription-polymerase chain reaction (RTPCR) testing was mandatory for all patients undergoing any Radiotherapy or CT procedures.

##### Radical radiotherapy:

- Patients requiring radical or curative therapy given top priority.
- Wherever possible hypofractionation schedule followed with or without simultaneous integrated boost (SIB).

##### Adjuvant radiotherapy:

- Priority given to those cases which belonged to high-risk groups like margin positivity or Extranodal Extension.
- For diseases where the risk of relapse was >20%, adjuvant RT was offered on priority.
- Low-risk cases kept under close observation.

##### Discontinuation of radiotherapy:

- Any patient who has discontinued the therapy and comes back for treatment after some time, they are first evaluated to exclude COVID-19 infection, days of break, the number of fractionation and dose received. A clinical examination is done to see the response to therapy.
- If the patient received more than half of the scheduled dose, the treatment was continued similarly without any change in the fractionation schedule. Gap correction was allowed where the gap is > 7 days.
- If the patient received less than half of the scheduled dose, the rest of the prescriptions were delivered in hypofractionation to the equivalent effective biological dose.

##### Brachytherapy:

- All patients for Brachytherapy are screened for COVID-19 infection. The procedure is done in an operation theatre with full PPE with separate sterilisation of instruments.
- Restricted only to Gynaecological malignancies. Brachytherapy for other sites like breast, head and neck abandoned.
- No change in the brachytherapy dose schedule was made.
- 9 Gy/2# schedule was allowed for patients with cancer of the uterine cervix and unable to come multiple times to the institute.

##### Palliative radiotherapy:

- Priority given for those patients who would have a substantial benefit (neurological cord compression, haemostatic RT, symptomatic brain metastases, pain not responding to opioids).
- Single fractionation 8 Gy is preferred. If multiple fractionations are indicated, 20 Gy/5# is the preferred dose.

##### Treatment review:

- Weekly review done for all on couch patients.
- Prophylactic nasogastric tube insertion avoided due to high aerosol contamination, if required, given with adequate PPE.
- Percutaneous endoscopic gastrostomy tube is the preferred prophylactic feeding tube before Radiotherapy to maintain proper hydration and nutrition.

##### Supportive therapy:

- Supportive therapy like dietary consultation, physiotherapy, swallowing exercises as instructed by the treating physician as far as possible.
- BT in few patients was a real problem during lockdown due to the unavailability of donors. Nevertheless, our blood bank ensured an uninterrupted supply of blood for cancer patients, wherever requested.

#### Changes in the CT schedule

The modifications of CT schedules were done adjusting the age and other co-morbid conditions of the patient (Tables 4 and 5). Because of travel restrictions during the lockdown, it was not easy to access hospital services.

##### Neo adjuvant:

- Dose intense schedules like triplet regimen CT were not chosen.
- Neo adjuvant CT was not done routinely, barring few subsites with modifications.
- Adults > 60 years not given infusion CT.
- Adults < 60 years with comorbidities provided with a 20% dose reduction after comorbidity adjustment.
- Continuous infusion CT was replaced with oral CT (Capecitabine in place of 5-Fluorouracil (5FU)) or hormonal therapy (ER+ve breast cancer) wherever possible.

##### Concurrent:

- There was a strong agreement not to abandon concurrent CT.
- Three weekly regimens changed to a weekly regimen.
- Patients who were > 60 years, where concurrent CT’s role shows no clear benefit not given concurrent CT.
- Concurrent CT was given only for mild hypofractionation (<2.4 Gy/fraction) or for conventional (2 Gy/fraction) Radiotherapy alone.

##### Adjuvant:

- Given to patients having a high risk of recurrences and diseases having a modest to a significant survival advantage.
- Three weekly regimens were adopted instead of weekly CT (e.g., weekly taxanes for breast cancer).

##### Palliative:

- Where expected survival is < 6 months, no palliative CT was done.
- For patients with expected survival > 6 months, palliation was done at home with oral CT.

#### Related to follow up

Cancer care requires much communication between the patient and the caregiver. A cancer patient needs to be followed up at regular intervals requiring multiple visits to the hospital. Due to lock down and no available transport, patients were not able to come to the hospital. During COVID-19 times, a large number of personal contact points were avoided, which could potentially minimise viral transmission. This communication gap leads to treatment interruptions during Radiotherapy and discontinuation of CT cycles, resulting in suboptimal care, cancer stage migration, ultimately leading to treatment failure and death. Patients were given the option of consultation from their homes through telemedicine, addressing cancer issues. A mobile application called ‘AIIMS Bhubaneswar Swasthya App’ was developed, which was available for patients to contact their concerned physicians directly. Even telemedicine helped the local doctors for the effective management of the patients in their homes.

#### Psychosocial issues

The most unforeseen and disheartening issue that arose due to the COVID-19 pandemic was the psychosocial issues that spared neither the patients nor the HCWs. There was a false sense of fear of isolation, uncertainty over the future, separation from family, economic slowdown and death. Patients and HCWs are labelled as a high risk for COVID-19 transmission. Many were asked to vacate their houses by housing societies and tenants. There was no availability of hotel rooms and guest houses due to lockdown, causing a great inconvenience to patients who have come for radiation therapy from far-off places. False news from social media was another cause of panic in this pandemic. HCWs and patients are aware of the disease’s facts and not to spread any rumours ascertained. For a cancer patient, fear of dying from cancer is far more than fear of contracting COVID-19. Understandably, they are reluctant to stay at home and instead want to see an oncologist, making them more vulnerable to contracting the disease.

## Conclusions

The COVID-19 pandemic arrived suddenly and was as surprising to patients as it was to oncologists. By the time both understood the magnitude of the problem, the government had imposed restrictions forcing the hospitals/departments to undertake tailor made changes to various treatment schedules. The authors have mentioned the changes made by the oncology department for the convenience of cancer patients. As India is in the peak of COVID-19, these types of modifications and modifications of treatment schedules will be the ‘New Normal’.

## Funding

This research did not receive any specific grant from funding agencies in the public, commercial or not-for-profit sectors.

## Conflicts of interest

The authors have no conflicts of interest to declare.

## Figures and Tables

**Table 1.** Composite data comparison during the lockdown (March 2020 to November 2020) versus same period and duration in 2019 (March 2019 to November 2019).

Period	Outpatient		Inpatient	External beam radiotherapy fractions delivered		Daycare			Brachytherapy
Lockdown period 2020 (March 2020–November 2020)	New	Follow up	1,281	Definitive	Palliative	CT	BT	SC	Fractions delivered
	2,095	8,082		3,630	368	3,605	143	362	123
	Total = 10,177			Total = 3,998		Total = 4,110			
Same period 2019 (March 2019–November 2019)	New	Follow up	1,116	Definitive	Palliative	CT	BT	SC	Fractions delivered
	5,263	15,102		10,438	559	6,069	311	581	183
	Total = 20,365			Total = 10,997		Total = 6,961			

CT, Chemotherapy/systemic therapy; BT, Blood transfusion, SC, Supportive care

**Table 2.** Changes in the radiotherapy fractionation schedule.

Protocol	Changes to radiotherapy schedule during COVID 19
Radical radiotherapy	Top priorityHypo fractionation with SIB
Adjuvant radiotherapy	Top priority where the risk of relapse > 20%Low priority to intermediate and low risks
Brachytherapy	7 Gy/3 fractions (preferred) and 9 Gy/2 fractions
Palliative	8 Gy/single fraction and 20 Gy/5 fractions
Treatment break due to lockdown	If received >50% of the total dose, the same fractionation schedule continued
	If received <50% of the total dose, the remaining treatment was rescheduled to hypofractionation wherever feasible

**Table 3.** Changes in radiotherapy protocol done according to disease site.

Disease site	Intention of treatment	Pre COVID19 radiotherapy fractionation	Changes made to radiotherapy fractionation during COVID 19
Head and neck	Adjuvant	60/66 Gy in 30/33 fractions	No changes
	Radical	70/60/56 Gy in 33 fractions	SIB 66/60/54 Gy in 30 fractions
	Palliative	30 Gy/10 fractions 40 Gy/15 fractions	20 Gy/5 fractions preferred 8 Gy/single fraction for haemostatic radiotherapy
Breast	Breast conservation therapy	40 Gy/15 fractions with boost of 12 Gy/5 fractions	No change
	Mastectomy	50 Gy/25 fractions	40 Gy/15 fractions
Glioblastoma multiformis	Age < 65 years Age > 65 years	60 Gy/30 fractions 60 Gy/30 fractions	No change 46 Gy in 16 fractions
Prostate	Radical	74–78 Gy/37–39 fractions	65 Gy/25 fractions
Rectum Lung	Neo adjuvant Radical	45–50.4 Gy/25–28 fractions 60 Gy/30 fractions	25 Gy/5 fractions 55 Gy/20 fractions
Palliative radiotherapy	Brain metastases Spinal metastases	30 Gy/10 fractions 30 Gy/10 fractions preferred. 8 Gy/single fraction	20 Gy/5 fractions 8 Gy/single fraction preferred

**Table 4.** Changes in the CT protocols.

Neo adjuvant	Dose dense and dose intense schedules abandoned
	Infusion CT replaced by oral therapy (e.g., oral capecitabine for 5FU)
	No infusion CT for patients age > 60 years
	Infusion CT at 20% dose reduction for patients age < 60 years with comorbidities
Concurrent	Considered for age < 60 years
	Abandoned for age > 60 years
	Three weekly regimens changed to a weekly regimen
	Administered for conventional (2 Gy) or mild hypofractionation (2.4 Gy) schedules only
Adjuvant	Priority for tumours with adverse prognostic features having high recurrence rates or modest to significant survival advantages
	Weekly regimen changed to 3 weekly regimens
Palliative	Expected survival < 6 months, no palliative CT is given
	Expected survival > 6 months of oral CT at home

**Table 5.** Changes in CT protocol according to site of disease.

Site of disease	Intention of treatment	Pre COVID 19 CT regimen	Changes in CT during COVID 19
Breast	Adjuvant CT	Weekly taxane	3 weekly taxane
Head and neck	Neo adjuvant CT	DCF regimen D1–D4 infusion	Taxane and carboplatin D1 infusion only
	Palliative CT	Cisplatin, 5FU/TPF /DCF regimen, metronomic therapy	Metronomic therapy with oral methotrexate and celecoxib preferred
Rectum/colon/stomach	Neo adjuvant/adjuvant/metastatic	FOLFOX/CAPOX regimen	CAPOX regimen/tab capecitabine only
